# Magnetic-Response-Driven Capture Behavior of Paramagnetic and Diamagnetic Fine Metal Particles in a Dry High-Gradient Magnetic Field

**DOI:** 10.3390/ma19010049

**Published:** 2025-12-22

**Authors:** Haozhou Chen, Huaiyu Wang, Osuke Miura

**Affiliations:** 1 School of Electrical and Information Engineering, Hunan Institute of Technology, 18 Henghua Rd., Zhuhui District, Hengyang 421002, China; chen_haozhou@163.com; 2 Department of Electrical and Electronic Engineering, Tokyo Metropolitan University, 6-6 Asahigaoka, Hino 191-0065, Japan; wang-huaiyu@ed.tmu.ac.jp

**Keywords:** dry high gradient magnetic separation, paramagnetic metals, diamagnetic metals, simulation analysis, Lorentz force, coefficient of restitution

## Abstract

Dry High-Gradient Magnetic Separation (Dry-HGMS) enables the manipulation of fine metal particles through their intrinsic magnetic responses. Research to date has predominantly addressed ferromagnetic powders, while the capture behavior of paramagnetic and diamagnetic particles with weak magnetic susceptibility has received limited examination. In this study, a multilayer magnetic filtration structure consisting of uniformly spaced unidirectional magnetic wires is developed to investigate the response-driven capture of such particles under dry conditions. By controlling the direction of the applied magnetic field, the system enables the selective capture of both paramagnetic and diamagnetic particles without inducing powder clogging. To clarify the capture mechanisms, a finite element method (FEM) framework is established that accounts for magnetic, drag, gravitational forces and Lorentz forces. The resulting capture maps reveal the dependence of particle trajectories on magnetic susceptibility, density, and electrical conductivity. Experiments performed on Al and Cr (paramagnetic) and Bi (diamagnetic) particles show trends consistent with the simulations. These results demonstrate that the proposed filtration system utilizes the magnetic-response characteristics of fine metal particles and extends the applicability of Dry-HGMS to weakly magnetic and diamagnetic materials.

## 1. Introduction

Over the past few decades, high-gradient magnetic separation (HGMS) has been widely applied in fields such as mineral processing [1,2,3], wastewater treatment [4,5,6], biosciences [7], and nuclear fuel recovery [8,9]. While particles generally exhibit good dispersion in water, fine particles dispersed in air tend to aggregate due to strong interparticle forces, particularly under dry conditions [10]. In dry HGMS systems, the packed magnetic matrix filters often provide numerous physical adsorption sites, causing magnetic and non-magnetic particles to adhere to its surface, which can lead to clogging and reduced separation performance [11].

In recent years, the demand for dry magnetic separation has been increasing. For example, carbon black powder used in the carbon electrodes of lithium-ion batteries in electric vehicles faces the risk of contamination by martensitic stainless steel particles during its production, which can cause safety hazards such as electrical sparks. Researchers have addressed various challenges in dry HGMS, focusing on optimizing magnetic matrix design [12,13], improving particle dispersion, and integrating multiphysical techniques [14,15,16]. However, most studies have concentrated on removing ferromagnetic powders, limiting the scope of application [17].

Meanwhile, recent studies have further explored high-gradient magnetic separation under different particle systems and operating conditions, including investigations on magnetic field distribution around magnetic matrices, capture behavior of weakly magnetic particles, and dry or quasi-dry separation processes [18,19,20,21].

An alternative approach for separating weakly magnetic or diamagnetic particles is magnetic separation using magnetorheological or ferrofluids, where the effective magnetic susceptibility of the medium is controlled by an external magnetic field. While such methods can enhance separation sensitivity, they rely on liquid media and introduce additional complexity in terms of fluid stability, contamination, and post-treatment, which limits their applicability in dry powder processing [22,23].

Therefore, further expanding the capability of dry HGMS without introducing auxiliary media remains of great practical importance. Recent efforts in this direction include the development of a novel multilayer magnetic filtration system featuring a rotating unidirectional magnetic fine wire structure, which effectively mitigates powder clogging issues and enables the capture of fine magnetic particles under dry conditions [24]. Additionally, our studies have employed thermally treated commercial SUS304 particles to fabricate fine metal particles with different magnetization intensities and identified the magnetic separation conditions for stress-induced martensitic transformation SUS304 particles [25]. Furthermore, the influence of eddy current effects on highly conductive, low-magnetization metal particles during the HGMS process has been analyzed [26]. However, these filtration systems are confined to vertical magnetic field configurations and are primarily designed for capturing paramagnetic and ferromagnetic particles. When attempting to capture diamagnetic particles, variations in particle positions on the magnetic fine wires alter the interaction between gravity and magnetic force, affecting the capture efficiency.

Building on these studies, in this study we propose a novel magnetic filtration system consisting of multiple layers of magnetic fine wires arranged with slight offsets along the same direction. By adjusting the magnetic field direction and intensity, the system effectively captures fine weakly paramagnetic and diamagnetic metal particles under optimized conditions. This study generates capture condition maps for such metal particles with varying densities and magnetic susceptibility, identifying the types of fine metal particles that can theoretically be captured using dry HGMS and demonstrating the potential of this technology for broader applications.

## 2. Principle of HGMS

**Figure 1:**
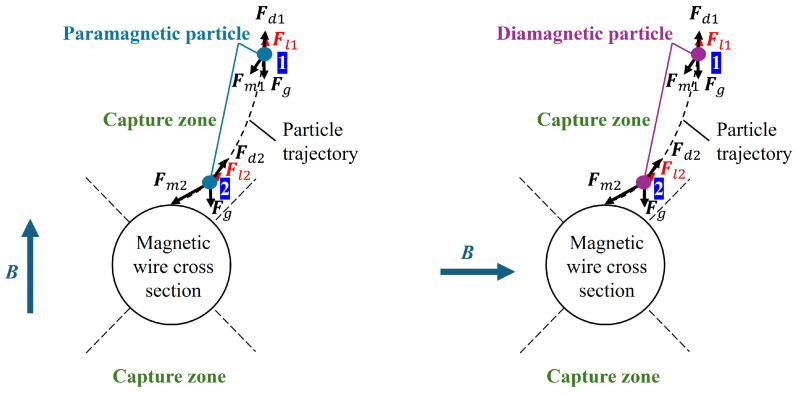
Various forces applied to a metal particle and the particle trajectory for paramagnetic (**left**) and diamagnetic (**right**) particles in HGMS system. The subscripts “1” and “2” indicate two representative positions of a particle along its trajectory [26].

In this study, the magnetic wire denotes a cylindrical soft-magnetic filament (SUS430) used as the magnetic matrix element in the HGMS system. Under an applied magnetic field, the wire produces a strong local magnetic field gradient around its surface, enabling the capture of fine metal particles. Figure 1 illustrates the forces exerted on a magnetic fine particle in the powder when it descends vertically toward a magnetic wire perpendicular to the magnetic field. When a magnetic field is applied perpendicular to a magnetic wire, a high magnetic field gradient is created around it, which creates a magnetic force that attracts magnetic particles. Then the forces acting on the particles at this moment include magnetic force, gravity, drag force, and Lorentz force, described by the following equations,

(1) $$F_{m}=\mu_{0}V_{p}M^{*}\cdot \nabla H$$

(2) $$F_{d}=6\pi \eta r_{p}\left(v_{f}-v_{p}\right)$$

(3) $$F_{g}=\left(\rho_{p}-\rho_{f}\right)V_{p}g$$

(4) $$F_{l}=\underset{V_{p}}{\;\int \int \int \;}J\times B\;dx\;dy\;dz$$

Here, $F_{m}$ represents the magnetic force, $\mu_{0}$ denotes the permeability of vacuum, $V_{p}$ is the particle volume, $M^{*}$ is an effective magnetic of particle, and H is the external magnetic field. $F_{d}$ represents the drag force, where η is the fluid viscosity, $r_{p}$ is the particle radius, $v_{f}$ is the fluid velocity, and $v_{p}$ is the particle velocity. $F_{g}$ accounts for gravity, $\rho_{p}$ and $\rho_{f}$ are the particle and fluid densities, respectively, g as the gravitational acceleration [27]. $F_{l}$ represents the Lorentz force, where J is current density of eddy current in particles and B is the magnetic flux density, The eddy currents considered here originate from particle motion in a spatially high gradient magnetic field, where the magnetic flux density experienced by the particle varies with time in its reference frame [26].

In previous works, for ferromagnetic particles $F_{l}$ is ignored because the magnetic force is much larger than it. However, for magnetic particles with weak magnetic susceptibility and high electrical conductivity, it was found that $F_{l}$ acts to suppress the motion of particles near the magnetic wire, making them more likely to be trapped on the magnetic filter [26]. Therefore, the particle trajectories are obtained by solving the time-dependent Newton’s equations of motion below.

(5) $$\rho_{p}V_{p}\frac{dv_{p}}{dt}=F_{m}+F_{d}+F_{g}+F_{l}$$

Additionally, in this simulation the coefficient of restitution ε was introduced because paramagnetic and diamagnetic particles with relatively weak magnetization are not necessarily captured after colliding with the magnetic wires.

## 3. Magnetic Filter and HGMS Simulations

### 3.1. Parallel-Moving Unidirectional Multilayer Magnetic Wire Filter

**Figure 2:**
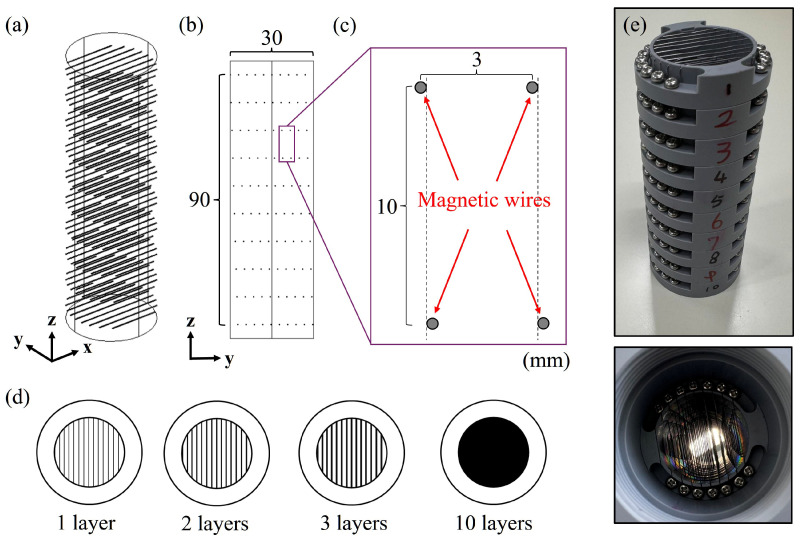
Structure and configuration of the parallel-moving unidirectional multilayer magnetic wire filter. (**a**) Filter shown in a 3-D perspective with a packing factor of 0.24%. (**b**) Filter shown in a 2-D perspective. (**c**) Enlarged view of the magnetic matrix, the structure with parallel-shifted layers. (**d**) The projection diagram of the filter, demonstrating that with 10 stacked layers, the magnetic wires fully cover the entire passageway. (**e**) Actual image of the magnetic filter.

Figure 2 shows the structure of a parallel-moving, unidirectional multilayer filter using SUS430 magnetic fine wires. SUS430 magnetic fine wires with a diameter of 0.29 mm are employed, and the spacing between wire centers is set to 3 mm. The cylindrical multilayer filter consists of 10 layers, each with a diameter of 30 mm and a height of 90 mm. Each layer contains 10 magnetic fine wires. The interlayer spacing is 10 mm, and magnetic wires for each adjacent layer is horizontally offset by 0.3 mm relative to the preceding layer.

In experiments, particles are allowed to fall freely from a position far enough away from magnetic wires. Immediately after fall, the magnetic field gradient is zero, so particles are influenced only by gravity and air resistance. As the particles approach the magnetic wires, they experience both a magnetic force and Lorentz force. When the magnetic field direction is parallel to the particle falling direction and perpendicular to the magnetic wire, the magnetic wire generates a negative magnetic field gradient, allowing it to trap paramagnetic particles. On the other hand, when the magnetic field, the magnetic wire, and the particle falling directions are mutually perpendicular, the magnetic wire generates a positive magnetic field gradient vertically upwards, allowing it to trap diamagnetic particles. The horizontal range of the initial position in which particles can be captured by the magnetic wires is defined as the Capture Range.

Based on the vertical projection of the multilayer filter, all particles moving within the filter can be captured if the capture range is sufficiently wide. Additionally, the volume filling ratio of the magnetic wires inside the filter is as low as 0.24%, effectively preventing powder clogging while maintaining a stable quasi-laminar flow state.

### 3.2. HGMS Simulation Method

In this study, the Lorentz force induced by eddy currents was incorporated into particle trajectory simulations using COMSOL Multiphysics 6.1.0.357 to evaluate the separation performance of the HGMS system. The simulations were performed in two steps. First, FEM calculations were conducted using the Magnetic Fields module to obtain the magnetic field distribution and field gradients around the magnetic wires. Second, particle trajectories were simulated using the Particle Tracing module based on the pre-calculated magnetic field. Particles were treated as independent, non-interacting entities, and interparticle interactions were neglected. Multiple trajectories were simulated by varying the initial particle positions and release conditions to analyze the capture behavior under different initial conditions.

**Figure 3:**
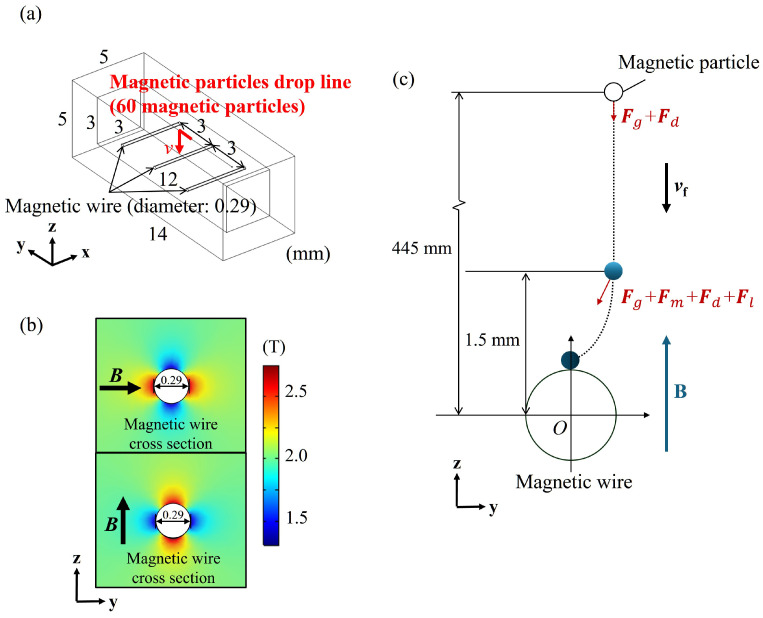
Simulation model and force analysis of particle capture around magnetic wires. (**a**) Simulation model of three magnetic wires. (**b**) Magnetic field distribution around the magnetic wires under a uniform 2 T magnetic field: horizontal orientation (upper) and vertical orientation (lower). (**c**) Simulation force state of a metal particle.

Figure 3a shows the structure of the three-wire filtration model that takes into account the influence of the adjacent magnetic wires. The model maintains the same wire diameter and center-to-center spacing as the actual filter in this study, measuring 0.29 mm and 3 mm, respectively. The medium for the simulation is air at room temperature. In the simulation of the capture for paramagnetic metal particles, the uniform magnetic field is set in the vertical direction. For the capture of diamagnetic metal particles, the magnetic field is oriented horizontally and perpendicular to the magnetic wires. Figure 3b shows magnetic field distribution around the magnetic wires at a uniform 2 T magnetic field with horizontal orientation (upper) and vertical orientation (lower).

The simulations use metal particles with a diameter of 60 μm, which is close to the average particle size of commercial metal powders used for real HGMS experiments. The physical parameters used in the simulations are listed in Table 1. A total of 151 metal particles, uniformly arranged with a spacing of 0.01 mm, are freely released from a position 445 mm away from the magnetic fine wires, initially influenced only by gravity and air resistance. As the particles approach approximately 1.5 mm above the fine wires, the magnetic field inhomogeneity becomes apparent, and the particles begin to experience both magnetic force and Lorentz force. Figure 3c shows the force state of a single particle.

In conventional analyses of magnetic particle capture using magnetic matrices, it is common to assume that a particle is captured upon contact with the matrix, setting the coefficient of restitution to zero. However, this approach has limitations in evaluating the capture of paramagnetic and diamagnetic particles.

Therefore, in this study, a coefficient of restitution was introduced for particle collisions with magnetic wires to enable a more precise assessment of the capture range.

### 3.3. Dry-HGMS Simulations Results

**Figure 4:**
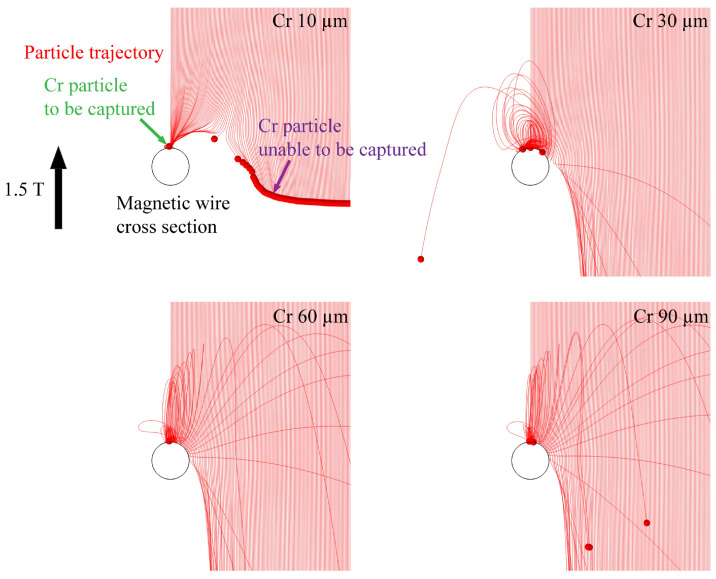
Dry-HGMS simulation images of Cr particles with different particle sizes, larger particles experience greater magnetic and gravitational forces, resulting in higher velocities upon collision. Consequently, their reflection distance increases, leading to a reduction in the capture range. ε = 0.8.

Table 2 and Table 3 indicate the material parameters used in the simulations [28]. Figure 4 presents examples of Cr particle trajectory simulations for particles ranging from 10 mm to 90 mm in diameter under a 1.5 T magnetic field, with a coefficient of restitution ε of 0.8. The coefficient of restitution ε characterizes the interaction between the magnetic wire and the particle, and depends on the material. In this simulation, ε was tested within the range of 0.1 to 0.8, and the results indicated that the capture outcome remained largely unaffected. Therefore, ε = 0.8 was assumed.

**Figure 5:**
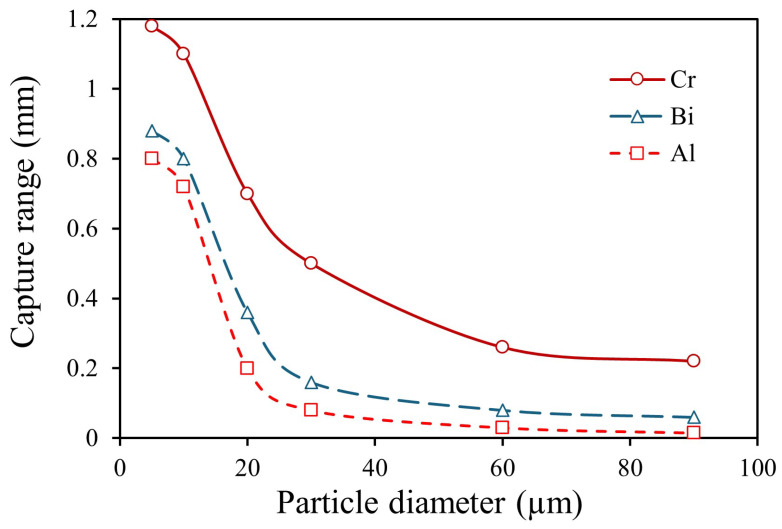
For paramagnetic metals Cr and Al, and diamagnetic metal Bi, it was shown that the capture range decreases as particle size increases.

Figure 5 illustrates the capture range of Cr, Al, and Bi particles with diameters ranging from 5 to 90 μm under a 1.5 T magnetic field. The simulation results indicate that, for the same metal, smaller particles are more easily captured. This can be attributed to the increased falling velocity of larger particles due to their higher mass, which reduces the time they are subjected to magnetic force. For different metals, particles with a higher absolute value of magnetic susceptibility and lower density tend to exhibit a larger capture range. This trend is explained by the stronger magnetic force acting on such particles and the longer duration for which they experience this force.

**Figure 6:**
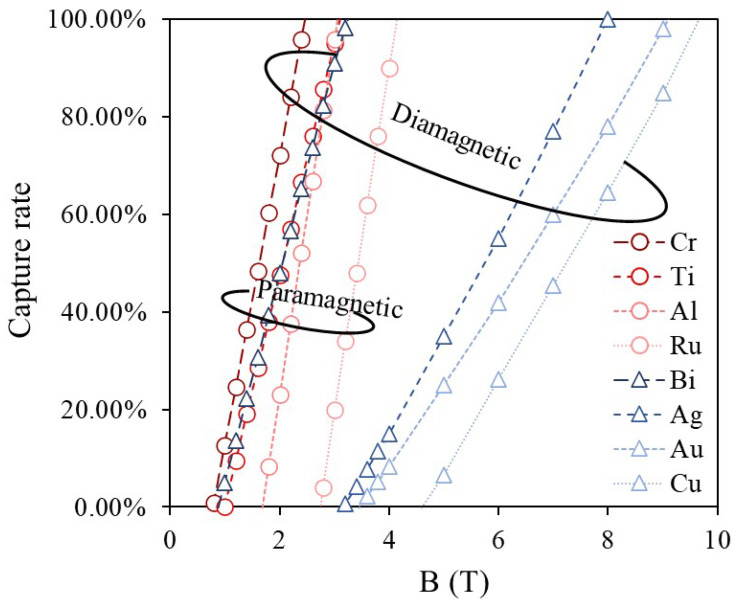
Magnetic field-capture rate simulations for paramagnetic and diamagnetic metal particles show that in general achieving 100% capture for diamagnetic particles requires significantly stronger magnetic fields compared to paramagnetic particles.

Figure 6 presents the magnetic field dependence of the capture rate for 60 μm paramagnetic and diamagnetic metal particles obtained from simulations. At low magnetic fields, gravity dominates over the magnetic force, resulting in a capture rate of zero. However, as the magnetic field increases, the magnetic field gradient around the magnetic wires becomes steeper, making the magnetic force the primary force acting on the particles and initiating their capture. Particles with higher magnetic susceptibility experience stronger magnetic forces, leading to a faster increase in capture rate and a higher likelihood of reaching a 100% capture rate. In general, since diamagnetic metals have lower magnetic susceptibility than paramagnetic metals, a stronger magnetic field is required for their capture.

**Figure 7:**
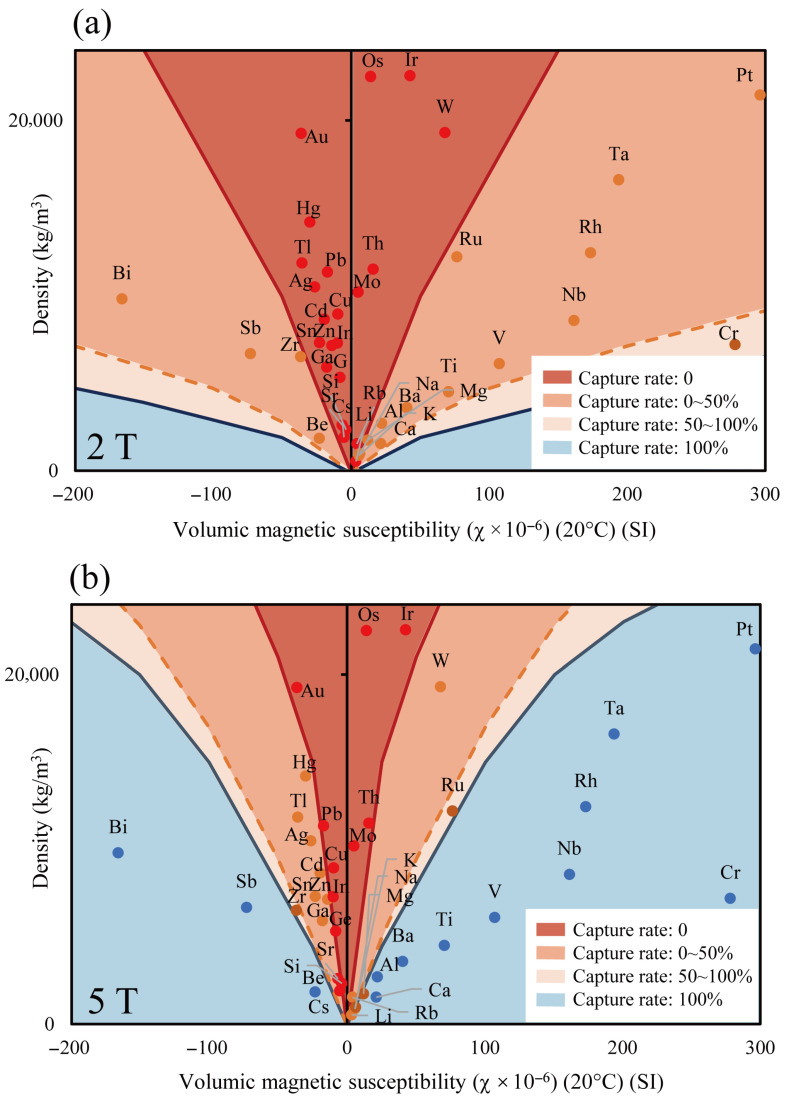
Distribution maps of the capture range for metal particles based on volume magnetic susceptibility and density under different magnetic fields. (**a**) 2 T magnetic field, (**b**) 5 T magnetic field.

Figure 7 shows the capture rate distribution of 60 μm metal particles under 2 T and 5 T magnetic fields, classified into four regions based on boundaries where the capture rate is 0%, 50%, and 100%. For particles of the same density, those with higher magnetic susceptibility experience stronger magnetic forces, resulting in an improved capture rate. Conversely, particles with higher density are more influenced by gravity, leading to a lower capture rate. Additionally, due to the small particle size of 60 μm, the effect of the Lorentz force is negligible [26].

At 2 T, no particles were fully captured, and only Cr particles exhibited a capture rate exceeding 50%, demonstrating that paramagnetic and diamagnetic metals are difficult to capture under weak magnetic fields. In contrast, at 5 T, the 100% capture region expanded, and the overall capture rate improved. Approximately half of the paramagnetic metals were fully captured, whereas diamagnetic metals exhibited lower capture rates due to their lower magnetic susceptibility. These results suggest that using this simulation method, capture rate maps can be generated for other materials based on their magnetic susceptibility and density.

## 4. Experiments of Dry-HGMS on Metal Particles

### 4.1. Experiments of Dry-HGMS on Paramagnetic Metal Particles

**Figure 8:**
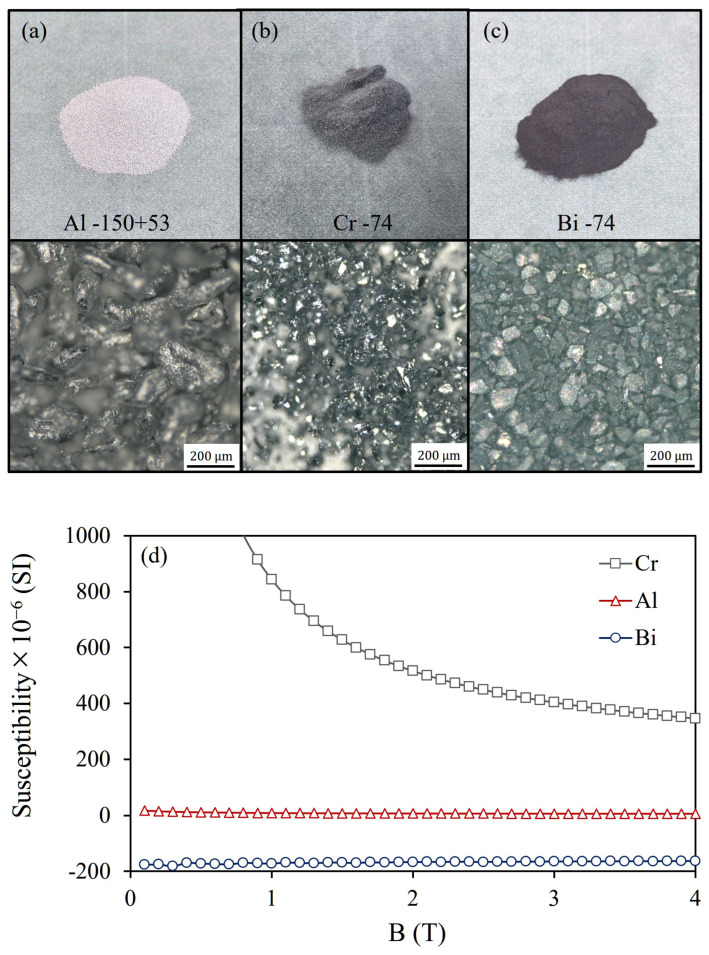
Samples used in the experiment and their microscope images: (**a**) Al −150 + 53 μm, (**b**) Cr −74 μm, (**c**) Bi −74 μm, (**d**) Magnetization (M–H) curves were measured using a SQUID magnetometer (MPMS, Quantum Design, San Diego, CA, USA).

Figure 8a,b present magnified microscope images and magnetization curves of the commercially available Al (−150 + 53 μm) and Cr (−74 μm) particles used in this experiment. The microscopic images were acquired using a digital microscope (VHX-X1, Keyence, Osaka, Japan) equipped with a VH-Z100R zoom lens. Here, the particle size range is denoted using the conventional sieving notation commonly adopted in powder and mineral processing. The expression “−150 + 53 μm” indicates particles that pass through a 150 μm sieve but are retained on a 53 μm sieve, corresponding to a particle size range of 53–150 μm. The Al particles exhibit an irregular granular morphology, and their volume magnetic susceptibility is slightly lower than the theoretical value due to partial surface oxidation. In contrast, the Cr particles, having undergone fine grinding, show slightly higher magnetization than the theoretical value due to enhanced surface effects (Figure 8d).

**Figure 9:**
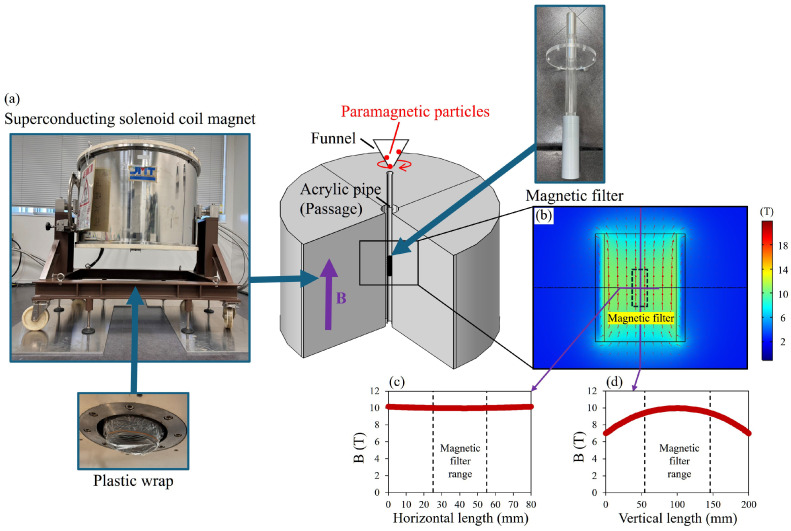
HGMS experiments using JMTD-10T100SS superconducting solenoid coil magnet (JASTEC, Kobe, Japan). (**a**) Equipment setup. (**b**) The magnetic field distribution around the magnetic wire filter when placed within the 2 T horizontal magnetic field generated by the apparatus. (**c**) The magnetic field in horizontal direction. (**d**) The magnetic field in vertical direction.

For the simulation experiments, the model described in Section 3 was applied, with relevant parameters listed in Table 4. Figure 9a illustrates the high-gradient magnetic separation experimental apparatus, which utilizes a superconducting solenoid electromagnet capable of generating a vertical magnetic field of up to 10 T. The magnetic field distribution is shown in Figure 9b–d. In the simulation experiments, the magnetic field is approximated as a uniform field. The effective structure of the magnetic filtration system is identical to the model in Section 3 and is positioned at the center of the electromagnet.

In the experiment, metal particles are introduced from a height of 445 mm above the magnetic fine wires using a vibrating hopper mechanism, with 0.50 g of particles supplied per batch. The particles undergo free fall, pass through the conduit, and are captured by the magnetic filtration system. Uncaptured particles are collected on a plastic wrap at the bottom, and their mass is measured using an electronic balance to determine the capture rate.

### 4.2. Experiments of HGMS on Diamagnetic Metal Particles

Figure 8c presents a magnified microscope image and magnetization curve of the commercially available Bi (−74 μm) particles used in this experiment. The Bi particles exhibit a distinct granular morphology, and their magnetization characteristics closely match the theoretical values.

**Figure 10:**
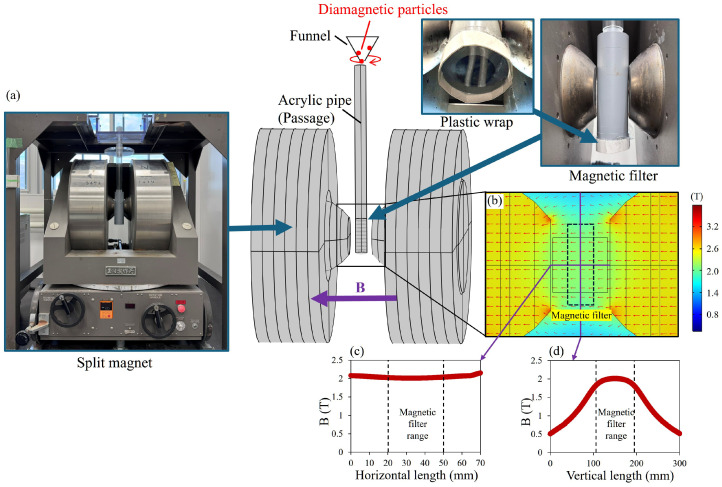
HGMS experiments using W-yoke-type fixed electromagnet (Tamagawa, Tokyo, Japan). (**a**) Equipment setup. (**b**) The magnetic field distribution around the magnetic wire filter when placed within the 2 T horizontal magnetic field generated by the apparatus. (**c**) The magnetic field in horizontal direction. (**d**) The magnetic field in vertical direction.

For the simulation experiments, the model described in Section 3 was applied, with relevant parameters listed in Table 2. Figure 10a illustrates the high-gradient magnetic separation experimental apparatus, which utilizes a split-type electromagnet capable of generating a horizontal magnetic field of up to approximately 2 T. The magnetic field distribution is shown in Figure 10b–d. In the simulation experiments, the magnetic field is approximated as a uniform field.

The experimental procedure follows the same method described in Section 4.1.

### 4.3. The Results of Metal Particles HGMS Experiments

**Figure 11:**
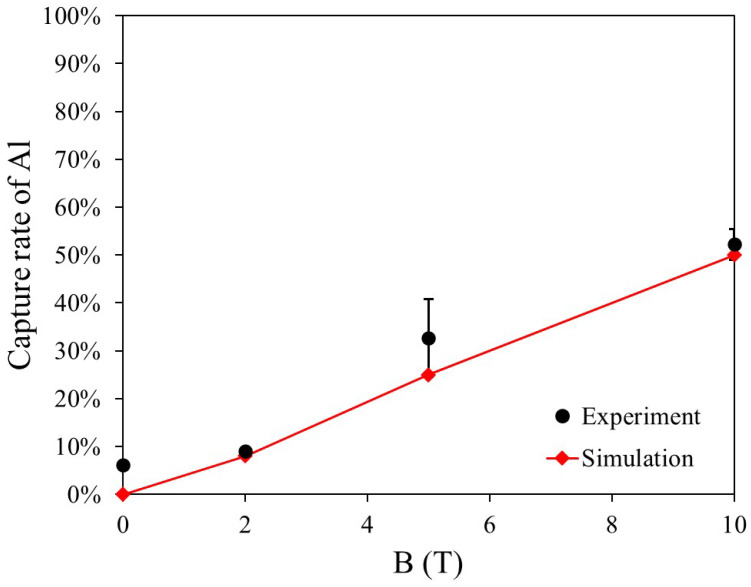
Comparison of experimental results and simulations for Al −150 + 53 μm.

Figure 11 presents the capture rate and simulation results for three dry HGMS experiments using 0.50 g of paramagnetic Al particles under a vertical magnetic field ranging from 0 to 10 T. In the simulation model, the particle diameter is set to 150 μm. The capture rate remains low within the 0–2 T range, and measurement errors arise due to some powder residues remaining inside the experimental apparatus. At 5 T, a significant discrepancy is observed between the experimental and simulation results, whereas at 10 T, the capture rates align well. This discrepancy at 5 T is likely due to the relatively weak magnetic force, making the capture process more sensitive to particle shape and other factors. In contrast, at 10 T, the magnetic force is sufficiently strong, leading to a stable capture rate of approximately 50%.

**Figure 12:**
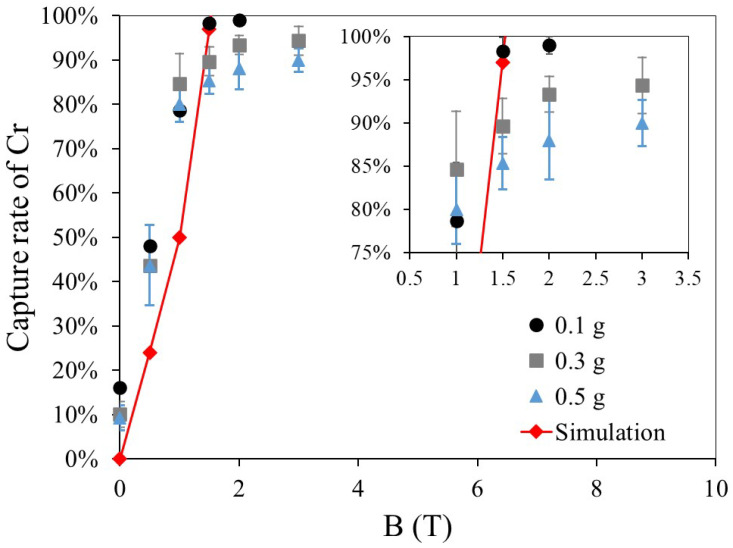
Comparison of experimental results and simulations for Cr −74 μm.

Figure 12 shows the capture rate and simulation results for three dry HGMS experiments using paramagnetic Cr particles of different feed masses under a magnetic field ranging from 0 to 3 T. In the simulation model, the particle diameter is set to 60 μm. At 0 T, approximately 10–15% of Cr particles remain inside the filter due to electrostatic effects and other interactions. According to the experimental results, the capture rate increases with the magnetic field strength up to 1 T, closely matching the simulation results. However, beyond 1 T, the capture rate increase slows significantly at a feed mass of 0.5 g. When the feed mass is reduced to 0.1 g, the experimental results align well with the simulation, suggesting that the filter has reached its capture capacity. To enhance this saturation limit, optimizing the filter structure by increasing the channel diameter or the number of filter layers would be effective.

**Figure 13:**
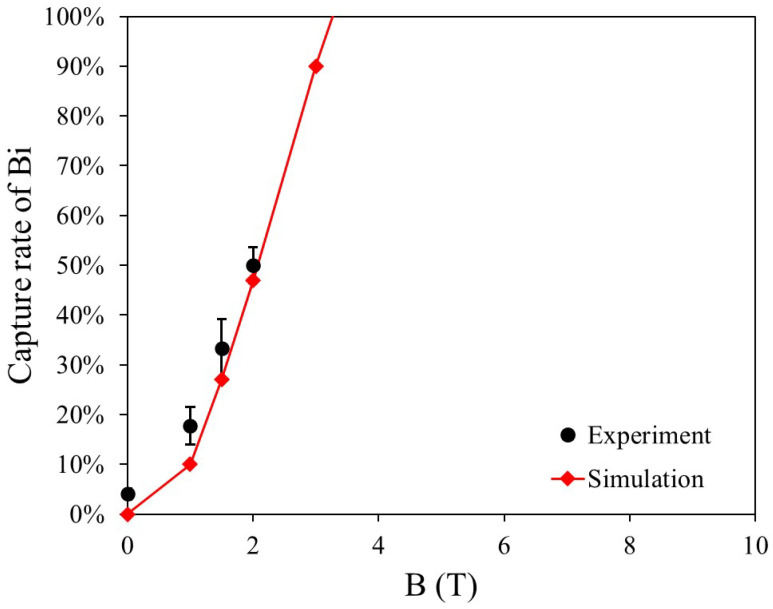
Comparison of experimental results and simulations for Bi −74 μm.

**Figure 14:**
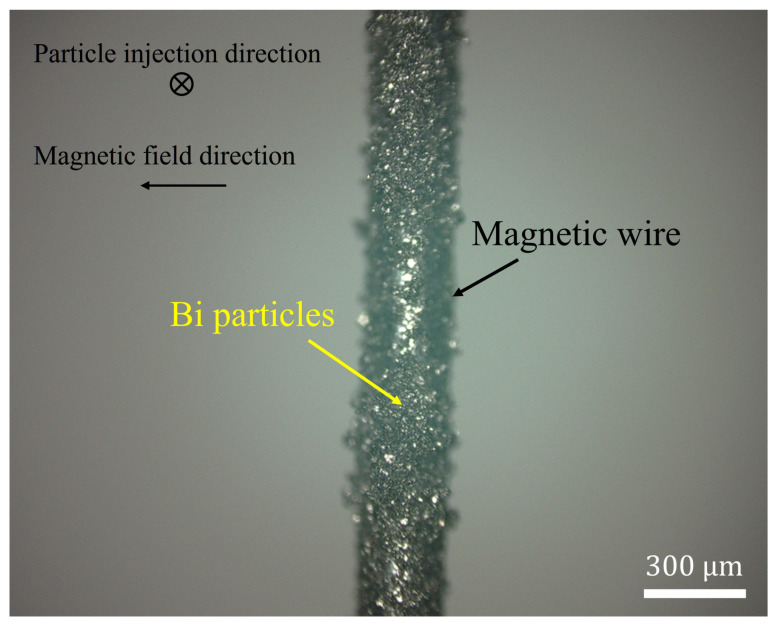
The post-experimental image of the Dry-HGMS experiment for Bi particles shows that the positions where the diamagnetic Bi particles were captured are perpendicular to the magnetic field direction.

Figure 13 presents the capture rate and simulation results for three dry HGMS experiments on diamagnetic Bi particles under a horizontal magnetic field ranging from 0 to 2 T. In the simulation model, the particle diameter is set to 60 μm. At low magnetic field strengths, some Bi particles remain in the filter due to electrostatic effects and other interactions. However, as the magnetic field strength increases, the magnetic force becomes dominant, causing the experimental results to approach the simulation predictions. Furthermore, the simulation results indicate that Bi particles can be fully captured at 3.3 T. Figure 14 presents a microscopic image of Bi particles adhering to the magnetic wire due to remanent magnetization after the experiment. The image confirms that the Bi capture region aligns with Figure 1.

## 5. Conclusions

In this study, a multilayer magnetic filtration system using parallel-moving magnetic wires was developed to construct a dry HGMS system. The system enables the efficient capture of both diamagnetic and paramagnetic metal particles under dry conditions without powder clogging. Its effectiveness was validated through numerical simulations and magnetic separation experiments. A newly developed FEM simulation incorporating the Lorentz force and the coefficient of restitution was used to generate capture range distribution maps for 60 μm metal particles based on their density and volume magnetic susceptibility. The results indicated that particles with a higher absolute volume magnetic susceptibility and lower density are more easily captured. The actual magnetic separation performance for paramagnetic metal particles was evaluated using aluminum (Al, −150 + 53 μm) and chromium (Cr, −74 μm) particles. In experiments conducted under a vertical magnetic field, a parallel-staggered filter was used for high-conductivity, low-susceptibility Al particles. Even at 10 T, the capture rate remained around 50%, suggesting that reducing the diameter and spacing of the magnetic fine wires could improve capture efficiency. In contrast, the Cr particle experiments showed that, at a feed mass of 0.5 g, the capture rate reached approximately 80%, indicating that the filter had reached its capture capacity. When the feed mass was reduced to 0.1 g, the experimental results closely matched the simulation predictions. To improve this saturation limit, optimizing the filter structure by increasing the channel diameter or the number of layers would be effective. Under a horizontal magnetic field, magnetic separation experiments were conducted using diamagnetic bismuth (Bi, −74 μm) particles. The results showed that at 2 T, the capture rate of Bi particles was approximately 50%. The experimental results closely matched the simulation predictions, confirming the accuracy of the simulation model. Furthermore, the simulation indicated that complete capture of Bi particles could be achieved at 3.3 T. This condition can be realized by generating a sufficiently strong horizontal magnetic field using a saddle-type superconducting coil.

Despite the fact that this study mainly focuses on spherical metal particles with a diameter of 60 μm, practical applications must also consider other factors, such as particle shape and electrostatic effects during the capture process. Furthermore, adjusting the diameter and spacing of the magnetic wires within a practical magnetic field range can further expand the complete capture range for fine metal particles in powder systems.

## Figures and Tables

**Table 1:** Parameters of the Magnetic Filament Model with Three Filaments.

Parameter	Value
Magnetic Wire Material	SUS430
Magnetic Wire Volume Magnetization	0.77 (SI)
Magnetic Wire Diameter	0.29 mm
Magnetic Wire Center-to-Center Distance	3 mm
Particle Diameter	60 μm
Particle Volume Susceptibility	−200 to $300\times 10^{-6}$ (SI)
Particle Density	0 to 23,000 kg/m^3^
Medium Material	Air
Medium Viscosity	$1.825\times 10^{-5}$ Pa·s
Medium Density	1.2041 kg/m^3^
Magnetic Field Strength	2 T; 5 T

**Table 2:** Parameter of Diamagnetic Metals.

Atomic Number	Metal	Density (kg/m^3^)	Conductivity ($\times 10^{6}$ S/m)	Volume Magnetic Susceptibility ($\times 10^{-6}$, SI)
83	Bi	9800	0.86	−166
51	Sb	6680	2.38	−73
40	Zr	6500	2.24	−37
79	Au	19,260	43.48	−36
81	Tl	11,850	6.02	−36
80	Hg	14,190	1.04	−30
47	Ag	10,490	62.50	−26
4	Be	1840	16.60	−23
50	Sn	7300	7.81	−23
48	Cd	8650	13.51	−20
31	Ga	5908	7.10	−18
82	Pb	11,340	4.85	−17
30	Zn	7130	16.89	−14
49	In	7280	11.11	−10
29	Cu	8930	59.77	−10
32	Ge	5323	0.22	−8
38	Sr	2600	4.39	−7
55	Cs	1900	4.76	−5
14	Si	2328	0.43	−4

**Table 3:** Parameter of Paramagnetic Metals.

Atomic Number	Metal	Density (kg/m^3^)	Conductivity ($\times 10^{6}$ S/m)	Volume Magnetic Susceptibility ($\times 10^{-6}$, SI)
3	Li	531	10.70	3
37	Rb	1530	8.00	4
42	Mo	10,200	17.54	5
19	K	870	14.58	6
11	Na	970	21.74	6
12	Mg	1740	25.64	12
76	Os	22,500	10.53	14
90	Th	11,500	5.37	16
20	Ca	1540	22.88	21
13	Al	2698	37.17	22
56	Ba	3580	2.00	40
77	Ir	22,550	18.87	42
74	W	19,300	18.18	68
22	Ti	4500	1.82	71
44	Ru	12,200	13.70	77
23	V	6100	3.85	107
41	Nb	8570	6.90	161
45	Rh	12,440	21.28	173
73	Ta	16,600	7.41	194
24	Cr	7190	7.75	278
78	Pt	21,450	9.43	296

**Table 4:** Parameters for Materials.

Material	Density (kg/m^3^)	Susceptibility $\times 10^{-6}$ (SI)	Conductivity $\times 10^{6}$ (S/m)	Particle Size Range (μm)
Al	2698	6	37.17	−150 + 53
Cr	7190	319	7.75	−74
Bi	9800	−166	0.86	−74

## Data Availability

The original contributions presented in this study are included in the article. Further inquiries can be directed to the corresponding author.
